# Editorial: Ionizing radiation reprograms tumor immune microenvironment by inducing immunogenic cell death

**DOI:** 10.3389/fimmu.2023.1235024

**Published:** 2023-06-20

**Authors:** Fei Yu, Kuangyu Shi, Shaoli Song, Haojun Chen, Weijun Wei

**Affiliations:** ^1^ Department of Nuclear Medicine, Shanghai Tenth People’s Hospital, Tongji University School of Medicine, Shanghai, China; ^2^ Institute of Nuclear Medicine, Tongji University School of Medicine, Shanghai, China; ^3^ Department of Nuclear Medicine, Inselspital, Bern University Hospital, University of Bern, Bern, Switzerland; ^4^ Computer Aided Medical Procedures and Augmented Reality, Institute of Informatics I16, Technical University of Munich, Munich, Germany; ^5^ Department of Nuclear Medicine, Fudan University Shanghai Cancer Center, Shanghai, China; ^6^ Shanghai Engineering Research Center of Molecular Imaging Probes, Shanghai, China; ^7^ Department of Nuclear Medicine and Minnan PET Center, The First Affiliated Hospital of Xiamen University, Xiamen, China; ^8^ Department of Nuclear Medicine, Institute of Clinical Nuclear Medicine, Renji Hospital, Shanghai Jiao Tong University School of Medicine, Shanghai, China

**Keywords:** ionizing radiation, tumor immune microenvironment, immunogenic cell death, immunotharapy, biomarker

Internal irradiation (IRT) and external-beam radiotherapy (EBRT) are two types of radiotherapy that use radiation beams like X-rays, gamma rays, or gamma rays to kill cancer cells directly and alter the tumor immunological microenvironment. Studies have currently shown a connection between radiotherapy and the tumor immune microenvironment, but further research is still required. In order to improve the efficacy of treatment, more information regarding the underlying mechanisms and how to improve the sensitivity of radiotherapy is required. Immune checkpoint blocking therapy (ICB) still has a low response rate due to the cold tumor microenvironment, which may be overcome so that the efficacy could be further increased when ICB is combined with other treatments. To assess the effectiveness and determine the prognosis, it will be helpful to search for the corresponding biomarkers.

As a result, the relationship between radiotherapy and the tumor immune microenvironment was revealed in the current Research Topic. Additionally, new techniques to increase the sensitivity of radiotherapy therapy were developed, and biomarkers to assess therapeutic efficacy and forecast therapeutic prognosis were investigated. The intricacy of the systemic and local immune environments in various cancer types is examined by Iliadi et al. How radiation shapes the immune landscape is focused on. Radiation was found to directly influence tumor cells and their surroundings, which mostly primes the immune response but may potentially inhibit it. When combined with immunotherapy, radiation causes an increase in infiltrating T cells and the production of programmed death ligand 1, which may be beneficial for the patient. Radiation causes an increase in immunosuppressive populations, particularly pro-tumoral M2 macrophages and myeloid-derived suppressor cells (MDSCs), in a number of malignancies. They also reveal potential prognostic and predictive information gleaned from the patient’s iTME and systemic immune profile to direct therapy choices. Zhu et al. found that the radio-sensitivity of TIME and circulating lymphocytes must be carefully taken into account when choosing the most appropriate radiotherapy regimen for combination with immunotherapy because different immune cell types, with different states of differentiation, exhibit different radio-sensitivities. The benefits of low-dose radiation (LDRT) over high-dose radiotherapy (HDRT) were highlighted by Ji et al. Firstly, LDRT has a low level of toxicity. It is challenging to reach the dosage limit for the organ at risk with SBRT, whereas dose restrictions will be easier to satisfy with LDRT, if radiotherapy is to be delivered simultaneously to many lesions within an organ. Secondly, LDRT is more secure for individuals who have previously undergone radiation treatment. When re-radiation is carried out with LDRT, there is a very little chance that the normal tissue dosage limitations will be exceeded. Finally, it is simpler to deliver LDRT. LDRT can be carried out in clinical settings using three-dimensional technology, but HDRT necessitates specialist imaging, respiratory gating, and even the implantation of gold fiducials.

The potential for radiotherapy to induce an immunogenic cell death (ICD) is supported by Mariampillai et al. They also demonstrate that using radiotherapy in combination with ATR inhibition boosts this potential. Additionally, they discovered that pan-caspase inhibition eliminates HMGB1 release, marginally boosts ATP secretion, and has little or no impact on ecto-CALR. Understanding these pathways will also probably make it easier to take advantage of the combined therapy’s immunostimulatory effects, perhaps by administering immune checkpoint blockade therapy afterward. Besides, Yuan et al. found that the gut microbiota may also be a common biological target for minimizing the side effects of radioimmunotherapy, inhibiting the target may improve efficacy and point patients with CRC in the right direction for achieving a longer survival and a higher quality of life after treatment. In order to achieve multimodal imaging and theranostics of lymph node metastasis, the Zhang et al. developed a nanoprobe conjugated trastuzumab based on upconversion nanoparticles, further developed a nanonuclear drug labeled ^68^Ga or ^177^Lu, and adopted a new imaging and theranostic strategy for lymphatic targeting. It is intended to direct research into and advancements in more potent theranostic methods for treating malignancies.

According to Gao et al., the parameters of ^18^F-fluorodeoxyglucose PET/CT served as biomarkers and were crucial in the treatment of ICIs, allowing for the assessment of the tumor microenvironment, the identification of immune-related adverse events, the evaluation of therapeutic efficacy, and the prognosis of the treatment. Transient receptor potential channels (TRPC) are essential regulators of the development and spread of cancer. Wang et al. created a unique risk score for colorectal cancer prognosis using eight TRPCG with excellent discrimination and calibration. A promising biomarker for ICIs and neoadjuvant therapy in the management of colorectal cancer could be the present TRPCRS. Fatty acid metabolism (FAM) is closely linked to the development of cancer and carcinogenesis.

In the context of cancer, Zhou et al. investigated the critical impact of FAM on the gut flora and metabolism. Patients with high-risk scores were closely associated with activating type I interferon response and inflammation-promoting actions, according to an extensive analysis of the tumor microenvironment based on the FAM-related signature. In patients with locally advanced rectal cancer, their findings suggest that FAM may be able to predict the outcome of neoadjuvant chemotherapy and radiation therapy as well as the prognosis of disease-free survival (DFS) and overall survival (OS). Finding precise biomarkers may aid in risk classification and treatment choices for locally advanced rectal cancer, according to Wang et al.

Altogether, after having seen all individual contributions to this Research Topic, we see that the topic of “*Ionizing Radiation Reprograms Tumor Immune Microenvironment by Inducing Immunogenic Cell Death*” is broad and has its roots in an active research field. The far-reaching implications of ionizing radiation-related tumor immune microenvironment make this an intriguing subject important for future studies to gain a better understanding.

## Author contributions

FY wrote the paper. All authors listed have made a substantial, direct and intellectual contribution to the work, and approved it for publication.

